# Preferred Methods of Measuring Work Participation: An International Survey Among Trialists and Cochrane Systematic Reviewers

**DOI:** 10.1007/s10926-022-10031-0

**Published:** 2022-03-26

**Authors:** Margarita Ravinskaya, Jos H. Verbeek, Miranda W. Langendam, Ira Madan, Suzanne M. M. Verstappen, Regina Kunz, Carel T. J. Hulshof, Jan L. Hoving

**Affiliations:** 1 Amsterdam UMC, Academic Medical Center, University of Amsterdam, Coronel Institute of Occupational Health, Amsterdam Public Health Research Institute, Amsterdam, The Netherlands; 2 Amsterdam UMC, Location Academic Medical Center, University of Amsterdam, Coronel Institute of Occupational Health, Amsterdam Public Health Research Institute, Amsterdam, The Netherlands; 3 Amsterdam UMC, Location Academic Medical Center, University of Amsterdam, Department Epidemiology and Data Science, Amsterdam Public Health Research Institute, Amsterdam, The Netherlands; 4 Guy’s and St Thomas’ NHS Trust and Faculty of Life Sciences and Medicine, King’s College London, Centre for Musculoskeletal Health and Work, London, UK; 5 Academic Unit EbIM, Evidence Based Insurance Medicine, Department of Clinical Research, University of Basel, Basel, Switzerland; 6 Centre for Epidemiology Versus Arthritis, Faculty of Biology, Medicine and Health, The University of Manchester, Manchester, UK; 7 NIHR Manchester Biomedical Research Centre, Manchester University NHS Foundation Trust, Manchester Academic Health Science Centre, Manchester, UK; 8 MRC Versus Arthritis Centre for Musculoskeletal Health and Work, University of Southampton, Southampton, UK

**Keywords:** Survey, Return-to-work, Worker participation, Vocational rehabilitation, Outcome studies

## Abstract

*Purpose* Heterogeneity in work participation (WP) outcomes measurements hampers large scale evidence synthesis in systematic reviews of trials. In this survey we explore authors’ reasons for choosing specific WP outcomes and their measurement methods, including employment status, absence from work, at-work productivity loss, and employability. *Methods* We contacted authors of 260 trials and 69 systematic reviews and asked closed and open-ended questions about previously used WP outcomes and measurement methods as well as their opinion on the best way to measure WP. *Results* In total, 91 authors from a wide range of professional backgrounds completed the survey. The majority of authors (86%) chose WP outcomes based on their use in previous similar studies. In most studies (88%), patients had not been involved in the process of selecting the WP outcome. Authors judged feasibility to be an important factor for choosing a measurement instrument (67%). Additionally, valid measurement tools should be available, easy to administer and not too time consuming. Although authors preferred registry data for long term follow-up, the availability and validity of registries was seen as a barrier. Most of the reviewers (72%) struggled to pool data because of variation in follow-up times and cut off points and varying definitions of work outcomes. Almost all (92%) respondents support the use of a Core Outcome Set for Work. *Conclusions* There is strong support from authors of trials and systematic reviews to develop a core outcome set on work participation outcomes for the evaluation of interventions.

**Supplementary Information:**

The online version contains supplementary material available at 10.1007/s10926-022-10031-0.

## Introduction

Authors of systematic reviews (SRs) state that inconsistent work participation outcome reporting in clinical trials hampers evidence synthesis in the field of occupational health [1–3]. Moreover, Cochrane review groups indicate that reliability and quality of SRs could be improved by the use of core outcome sets [4]. However, to date there has been no agreement on a core outcome data that should be collected in health or occupational research. In light of this, we set up an international collaboration to develop a Core Outcome Set for Work Participation (COS for Work), using the Core Outcome Measures in Effectiveness (COMET) Trials approach (http://www.comet-initiative.org/studies/details/1195).

As a first step in the development of COS for Work we performed a systematic review and showed the variety of work participation outcomes currently used in trials [5]. We showed that publications from trial authors report little or no rationale to justify specific outcomes or measurement instruments over another, including supporting information to use specific questionnaires or specific time points. Understanding why authors of trials choose certain outcomes or measurement instruments would help inform consensus on choosing suitable methods to measure work participation across trials. In addition, having the opinion of SR authors on work participation outcomes and involving them in the development of a COS is important as consistent outcome measurement is critical for synthesizing results from different trials [6].

The overall aim of this survey was to explore researchers’ perspectives and experiences when considering (the measurement of) work participation outcomes for RCTs and SRs. The specific objectives were (1) to describe the factors that RCT authors and systematic reviewers consider when choosing work participation outcomes, measurement tools and time-points, (2) to enquire about the experience with outcomes and measurement methods that authors of RCTs and reviewers have used previously, and (3) to determine what advantages and barriers researchers foresee in using a COS for Work.

## Methods

### Recruitment of Survey Participants

We identified RCT authors (short ‘authors’) and Cochrane systematic reviewers (short ‘reviewers’) in two ways. The RCT authors were identified based on trials reporting paid work participation through searches in Medline, Embase, PsycINFo and Cochrane Central between 01/01/2014 and 21/05/2019, as part of a systematic review [5]. From the 436 trials identified we included all RCTs that included vocational interventions aiming to directly improve work participation (n = 71). Furthermore, we randomly selected twice as many non-vocational RCTs (n = 142) since we expected a lower response rate within the non-vocational authors’ group. In addition, we contacted the corresponding author of all systematic reviews published by the Cochrane Work Group on a work participation topic in the last five years (2017 to 2021).

### Development of the Survey Content and Building the Questionnaire

We identified which topics should be included in the survey based on our systematic review on heterogeneity of work participation outcome measurements [5] and iterative discussions with our research team. First, we grouped the outcomes of this review into four main outcome categories based on a study by Verstappen et al. [7]; “employment status, “absence from work”, “at-work productivity loss” and “employability” (see definitions of the four categories in Online Appendices 1 and 2). Within these categories we explored heterogeneity based on: (1) the terminology describing the measured outcomes, (2) data collection methods; (clinimetric properties of) tools, questionnaires, registries. (3) follow-up times and (4) cut-off points; e.g. after what point in time *employment* or *return to work* is present. Questions in this survey are aimed to gain insight into why such extensive heterogeneity currently exists. Next, survey questions were added from the literature on methodology and existing issues in work participation outcome measurements [8–14]. Finally, we held several discussions within our research team to make a selection of the most important questions for inclusion within the survey.

Two types of answer options were used. Closed ended questions on the topic of work participation could be answered with a yes or a no. In addition, using open ended questions, we also asked participants to elaborate on answers such as titles of most favored questionnaires, or why a certain method was preferred.

### Survey Topics

The first survey section of the questionnaire was on demographic characteristics, the professional experience of the respondents and the field of expertise. Furthermore, the survey included the following topics:

- Reasons for choosing work participation outcomes and measurement methods in the protocol stage
- Reasons for which outcomes should be primary or secondary (if at all)
- Prior experience with the chosen outcomes and measurement methods
- Follow-up times
- Familiarity with the use of a COS
- Expected barriers, advantages and recommendations for the development and implementation of COS for Work
- Whether certain types of outcomes should be included in a COS
- Whether certain outcomes or measurement methods should not be considered for a COS
- Interest in participation as a stakeholder in the consensus process during the development of COS for Work

The questions for authors and reviewers contained the same topics except for two aspects: the authors were asked questions that reflected on their experience with measurement methods (for the whole survey, see Online appendix 1), and the reviewers were asked questions on their experience with data synthesis (see Online appendix 2).

### Pilot Test

First, we tested the survey amongst eight authors and reviewers for clarity, completeness and burden and revised the survey using their feedback. The pilot participants were co-authors of this survey and colleagues from the Coronel Institute of Occupational health (Amsterdam UMC, Amsterdam, The Netherlands) who co-authored an RCT or review on work participation during the past 5 years.

### Survey Administration

Each survey participant was requested to complete an online questionnaire (using LimeSurvey) about their RCT or review which was included as a PDF file attached to the e-mail invitation. The participants were advised in the invitation letter that the survey should take about 20 min to complete. We first used the e-mail address of the corresponding RCT author or reviewer provided in the manuscript. If unsuccessful, we emailed the first, second or last author. All review authors which authored a Cochrane review in the past five years received an invitation. Non-respondents received in total four weekly reminders.

### Analysis

Data were converted from LimeSurvey to the SPSS format, analysis was performed in SPSS.

#### Closed-Ended Questions

We used descriptive statistics to summarize categorical responses in absolute frequencies and percentages. To establish whether authors and reviewers differed in their answer patterns to identical questions we performed Chi-square tests of independence. We tested two-sided with an alpha of 0.05 without correction for multiple testing.

#### Open-Ended Questions

Open-ended questions which offered additional insights to the closed-ended questions were analysed inductively by two researchers (M.R, J.V.) separately in Microsoft Excel. We identified common themes, assigned codes to all the comments and used an Excel pivot table to establish which topics/codes were the most to least frequently mentioned. In addition, we selected illustrative verbatim quotes to illustrate a theme.

## Results

### Recruitment and Characteristics of Participants

The survey was sent to 260 authors and 69 reviewers, 99 replied, 71 authors (response rate 27%) and 28 reviewers (response rate 41%). Participants resided in 21 countries across all continents. Their main professional background varied from vocational health to medical, social and psychological sciences and health economics, their mean experience in research using work participation outcomes was 14 years (mean, SD 9) ranging from 1 to 39 years.

### Types of Work Participation Outcomes

Outcomes indicated by authors and reviewers as the main outcome of their study were grouped in the following four pre-determined categories: employment status (n = 13, 14%), absenteeism and return to work (RTW) (n = 52, 57%), at-work productivity loss (n = 21, 23%), and employability (n = 2, 2%). Four authors (4%) did not respond to this question.

### Reasons for Choosing Work Participation Outcomes

#### How Work Participation Outcomes Were Chosen

The majority of respondents (79/91, 87%) based the choice of their study outcomes on the outcomes’ previous use by researchers in similar studies, followed by the relevance of the outcome to the health problem (70/91, 76%) and aims of the interventions (64/91,70%) (Table 1). To a lesser extent the choice was based on consulting stakeholders such as funders, policy makers and health professionals (27/90, 30%).

**Table 1:** Reasons for choosing specific work participation outcomes in a recent RCT or review

Work participation outcomes were chosen, based on … (n = 91)	Agreement with statement % (n)
• … their use in similar studies	87% (79)
• … relevance to the health problem of the study population	76% (70)
• … the anticipated impact of the intervention(s)	70% (64)
• … their previous use by the authors	64% (59)
• … pre-specified measurement methods (n = 90)	38% (35)
• … the consultation of stakeholders such as funders, policy makers, health professionals (n = 90)	30% (27)
• … a core outcome set (n = 90)	18% (16)
• … a theoretical framework/conceptual model/logic model (n = 90)^a^	17% (15)
• … a consensus process (e.g. Delphi study) of experts or an advisory group	16% (15)
• … a specific stakeholder perspective (n = 90)	14% (13)
• … consultation of patients/consumers (n = 90)	12% (11)

^a^Unless indicated otherwise, questions were answered by 91 respondents

Quotes from the authors in the open ended questions also mention perspectives of the employer, employee and society (including reflections on cost-effectiveness and social insurance) without any mention of involvement of these stakeholders in the selection of outcomes. Patients/consumers were barely mentioned in this decision process by the authors.

Authors only infrequently reported theoretical models to underpin why certain outcomes were chosen (15/90, 17%). The authors who said to have used a theory or model and elaborated their answer in the open fields mentioned the following amongst others: the International Classification of Functioning Disability and Health [15], the Karasek Model [16], the Work Ability Theory [17] and psychosocial theories [18–22]. In addition, national policy guidelines, and labour economic theory were also mentioned to influence the choice of outcomes. Authors (16/90, 18%) infrequently indicated that they used core outcome sets. However, analysis of the open ended questions showed that most answers specified theories and measurement instruments rather than a core outcome set. Nonetheless, three specific core outcome sets/ guidelines were used for three different studies: low back pain [23], rheumatoid arthritis [24] and chronic pain [25].

#### How the Distinction is Made Between Primary and Secondary Work Participation Outcomes

Distinctions between primary and secondary work participation outcomes mostly depended on the validity of an instrument (30/63, 48%) (Online Appendix 3). The choice for outcomes was infrequently determined by stakeholders within the RCTs (18/64, 28%) or reviews (5/26, 19%).

The open ended questions revealed that the availability of data from registries was also considered a factor in the selection of outcomes as illustrated by one participant: *In this study we aimed at using both health insurance data and data from the employment office. Neither measures actual "RTW", but they were suitable for the present study. However, the data from the employment office turned out not to be reliable. Thus our primary outcome was based on data from health insurance data.* Furthermore, open ended questions also revealed employer and economic perspectives were a reason to choose a work participation as a primary outcome primary.

#### Pre-specification of Work Participation Outcomes

Table 2 describes whether authors pre-specified their work participation outcomes. According to the authors and reviewers, the majority (66/90, 73%) of work participation outcomes were defined in a protocol, although some (28/89, 31%) were explored post-hoc. RCT authors pre-specified primary versus secondary outcomes and methods of analysis less frequently than reviewers (69% of authors vs. 92% of the reviewers, p = 0.018).

**Table 2:** Routines to specify work participation outcomes prior to initiating the RCT or review

Pre-specification of work participation outcomes	Agreement with statement		
(n = 90)	All % (n)	RCT authors % (n)	Reviewers % (n)
Respondents would use the same work participation outcome in the future (n = 89)	84% (75)	84% (54)	84% (21)
A protocol described the analysis plan for all work participation outcomes (n = 89)	74% (66)*	69% (44)	92% (24)
A protocol defined which work participation outcome was primary and which one was secondary	73% (66)^a^	69% (44)	92% (24)
All work participation outcomes were analyzed and reported as planned	38% (34)	84% (54)	92% (24)
Some work participation outcomes were not pre-specified but explored post-hoc (n = 89)	31% (28)	34% (22)	23% (6)
Work participation outcomes were documented in a trial register	–	65% (40)	–

If not indicated otherwise, questions were answered by 90 respondents, 64 authors and 26 reviewers

(–): Reviewers did not receive this question

*Difference between RCT and SR authors p < 0.05

### Measurement Methods

#### Reasons for Choosing Measurement Instruments in RCTs

Reasons for choosing measurement instruments, such as their validity and feasibility, are described in Table 3. Authors mainly selected the measurement instrument so that it could be used for effectiveness evaluation at baseline and follow-up (45/57, 79%). The second most important factor was the feasibility of the measurement method/instrument (38/57, 67%), followed by the validity (28/57, 49%), reliability (23/57, 40%) or responsiveness (17/57, 30%) of a measurement instrument. Almost a quarter of the authors self-developed the items of their measurement instrument.

**Table 3:** Reasons for choosing a measurement method/instrument in RCTs

Reasons for choosing a measurement method/instrument in RCTs N = 57	RCT authors’ agreement with statement % (n)
The measurement method was described in a protocol	91% (52)
Important that the instrument could be used at baseline and follow-up	79% (45)
The choice of the measurement instrument was based on (more than one answer choice allowed)	
• feasibility	67% (38)
• high validity	49% (28)
• high reliability	40% (23)
• high responsiveness	30% (17)
Self-developed items were used	23% (13)
An instrument that could show composite outcomes was chosen	19% (11)
Legislative, social insurance factors determined the choice	16% (9)
Adapted items based on an existing questionnaire were used	14% (8)

Answers to open ended questions indicated the importance of feasibility in terms of ease of data gathering and administration. Feasibility was described in terms of time and ease of completing the self-report questionnaires by patients, as illustrated by a respondent: *most published instruments to evaluate impact of health on worklife ask too many questions for an RCT in seriously ill patients*. *While we looked for a well-developed, and extensively validated tool, we also needed a practical tool for collecting over a 6 month clinical trial as an exploratory endpoint.*

#### Challenges with Pooling Work Participation Outcomes

The majority of reviewers experienced challenges with pooling work participation outcomes from different RCTs (18/25, 72%), mostly due to varying cut-off points and follow-up times (20/25, 80%) and heterogeneous definitions of outcomes in the RCTs (14/25, 56%) (Online Appendix 4), as illustrated by elaborations from the open ended questions: *RTW included return to either full or part-time employment, to the same or a reduced role and to either the previous job or any new employment*. … *Different definitions of paid work, especially paid work with some financial benefits of social security institutes for employers*. In addition, reviewers mentioned challenges comparing results and measurement methods which were described ambiguously.

#### Preferred Ways to Measure and Report Work Participation Outcomes

The majority of reviewers preferred *work status* outcomes measured as duration of employment in work days (17/25, 68%) (Online Appendix 4). For *absenteeism*, reviewers found that measures should include sick leave duration in work days (21/25, 84%), the proportion of sick listed employees (13/25, 52%) and sickness leave spells (10/25, 40%). Reporting of *At-work productivity loss* should include mean and standard deviation (14/25, 56%), mean differences (52%), or standardized mean differences (12/25, 48%) and confidence intervals (11/25, 44%). We did not ask the reviewers whether any of the proposed measures should be reported exclusively or simultaneously.

In an open ended question we asked authors about their preference for self-reported questionnaires or registry data for long term follow-up. About half of the responding authors (19/37, 51%) preferred registry data with reasons such as decreasing the risk of recall bias. However, open ended questions revealed authors were also cautious about the reliability, feasibility, and validity of registers as stated by two authors: *Depends on how reliable the registries are and if they are accessible or Registry data give you the opportunity for long-term follow-up, but a major limitation is inaccuracy about diagnosis and diagnosis for sick leave may change over time.*

#### Follow-up Time

Across all outcome categories authors (n = 55) and reviewers (n = 25) preferred follow-up measurements for up to 12 months, and, to a lesser extent, up to 6 or 24 months (Table 4). Long term outcomes of 24 months or more were more frequently chosen for work status and absenteeism compared to the other two outcomes.

**Table 4:** Proposals for ideal follow-up times for different work participation outcomes

Ideal follow-up times N = 80	“Work status” outcomes “YES” %, (n) respondents	“Absenteeism” outcomes “YES” %, (n) respondents	“At-work productivity” outcomes “YES” %, (n) respondents	“Employability” outcomes “YES” %, (n) respondents
1 month	8% (6)	13% (10)	11% (9)	8% (6)
3 months	19% (15)	24% (19)	19% (15)	16% (13)
6 months	28% (22)	30% (24)	26% (21)	21% (17)
12 months	45% (36)	44% (35)	43% (34)	40% (32)
24 months	35% (28)	29% (23)	21% (17)	21% (17)
> 24 months	23% (18)	23% (18)	14% (11)	19% (15)

Reported are the number of respondents who agreed with each proposed duration of follow-up time

As illustrated by open ended questions, duration of follow-up should depend on the population/health problem and the type of intervention as illustrated by a respondent: *…. depends on the objectives and context of the study (i.e., clinical settings, disability outcomes…) 3-, 6- and 12 months are good for clinical settings/clinical muskuloskeletal conditions. 3-,5-year are better for economic impact of MSK condition studies.*

### Support for a Core Outcome Set for Work Participation

Almost all authors and reviewers (92%, 89%) support the use of a COS for work participation (Table 5) to facilitate the comparison of work participation data on a large scale. To be included in a COS for work participation authors and reviewers supported the inclusion of work status and absenteeism somewhat more frequently than at-work productivity loss outcomes and employability outcomes (respectively 98%/93% versus 56%/64%). The most frequently reported anticipated barrier for a generic COS was that social benefit and health care systems may require different effect measures (68/78, 87%).

**Table 5:** Implementation of a generic COS for work

Support for the implementation of a generic COS for Work (n = 78)	Agreement with statement		
	All %, (n)	Authors %, (n)	Reviewers %, (n)
I would support the use of a generic COS for Work	92% (70)	89% (48)	92% (22)
A COS for Work should include at least …			
… work status outcomes	98% (77)	98% (53)	100% (24)
… absenteeism outcomes	93% (73)	93% (50)	96% (23)
… at-work productivity loss outcomes	66% (52)	63% (34)	75% (18)
… employability outcomes	64% (50)	59% (32)	75% (18)
Barriers for a COS for work			
Social benefit and health care systems require different effect measures	87% (68)	91% (49)	80% (19)
I would choose a disease specific COS if it is available	11% (9)	13% (7)	8% (2)
In my health field it is not possible to use a measurement instrument which is not disease specific	1% (1)	2% (1)	0
In my health field, work related definitions, outcomes, i.e. “what” should be measured is decided and cannot be revised	2% (2)	2% (1)	4% (1)

In the open ended questions respondents elaborated on other barriers such as a COS that could be too generic, or not be feasible enough if it included too many outcomes. In addition, respondents saw a challenge in making the COS known across all research fields, for example in clinical research. Furthermore, respondents anticipated some reluctance of researchers to change their familiar and previously used methods. To mitigate the reluctance to use of a COS other factors such as ease of use, no additional costs and clear added value should be considered.

## Discussion

### Summary of Main Findings

This survey shows researchers have different perspectives and experiences when considering work participation outcomes, measurement methods and time-points in RCTs and SRs. Choosing work participation outcomes not only depend on their relevance for the health problem or anticipated impact of the intervention, but researchers particularly prefer outcomes if they were used in similar studies. Choosing a measurement method like a specific questionnaire or data registry was informed by several reasons, feasibility being an important reason, next to other clinimetric properties.

In addition, although registry data are considered relevant for long term follow-up, authors are also cautious about the availability, validity and comparability of registry data across countries. There is a need to establish consensus on how to measure work participation outcomes and uniform terminology, specifically in terms of employment and return to work. This survey shows the development of core outcome sets for work is highly supported by RCT authors and systematic reviewers. COS development should take into account possible barriers for its acceptance, adaptation and use.

### Strengths

The main strength of this international survey is that researchers from vocational, medical, clinical, social and economic fields were asked on their views on measuring work participation. Given its wide spread across countries and disciplines we consider the results to be generalizable within the field of occupational health. So far we are unaware of any similar survey to date on this topic. The benefit is that the survey’s findings are based on empirical knowledge of potential end-users of a COS for Work. Relevant stakeholder involvement is crucial for adequate development of a COS [14]. By considering opinions of RCT authors and systematic reviewers we ensure that opinions of these two types of end users of the COS can be taken into account during the following steps of COS development. This survey provided us with in-depth information in addition to our previous work describing how work participation is measured internationally [5]. We also provided an opportunity for qualitative information from open ended questions to grasp the reasoning behind the choices that authors and reviewers make in terms of methods and outcome selection.

### Limitations

A limitation of this study is that the response rate was not high (28%) but this is similar in most online surveys [26]. It might be that the high non-response has resulted in a selection bias whereby authors who have a positive attitude towards a COS were more likely to respond. It was not always clear if the respondents understood our prespecified answer statements in the questions. For example, the question on whether authors had used a COS showed only a few understood what a COS was, as the replies included questionnaires and theories rather than a COS. However, we think most other questions were formulated rather unambiguous.

### Results Compared to Previous Findings

Our findings confirm that outcomes are chosen based on the type of health problem [27–29]. Moreover, as in previous studies [5, 30, 31] we found that data on work participation outcomes are difficult to combine due to lack of varying terminology. Specifically, reviewers indicated that they struggled with combining sickness absence and RTW data due to unclear reporting, varying cut-off points and lack of consensus on what counts as RTW. Moreover, the foreseen barrier of the comparability and validity of registry data is backed by literature [32]. A difference of our study with findings of a scoping review by Arienti et al. [33] is their finding of a lack of pre-specification of rehabilitation outcomes. Authors in our survey indicated that in most cases outcomes are pre-specified which may be explained the increasing publication of RCT study protocols [34].

Last, our results are in line with EULAR recommendations that both aggregated measures such as sick leave days as well as the proportion of sick listed employees are important outcomes to consider [35].

### Implications for Practice

The most important implication for practice is that the majority of authors who responded to our survey would welcome a generic COS for Work and would support its use. The findings of this survey will be used for the development of a COS for Work. Although rarely involved in determining which work outcomes are measured, we think involvement of stakeholders is a crucial step to ensure the inclusion of relevant outcomes and promotion and dissemination of a COS [6]. This may be especially important to consider in the development of the COS, as literature shows that in vocational rehabilitation what is most important to measure differs from the employer, employee, societal and health providers perspectives [13, 36, 37]. In the next consensus phase of our COS for Work we will ensure the inclusion of a diverse range of stakeholders.

## Supplementary Information

Below is the link to the electronic supplementary material.

Supplementary file1 (DOCX 51 kb)
