# Supplementary material for: Preferred Methods of Measuring Work Participation: An International Survey Among Trialists and Cochrane Systematic Reviewers
Source: J Occup Rehabil. 2022 Mar 26;32(4):620–8. doi: 10.1007/s10926-022-10031-0 (PMC9668767; doi:10.1007/s10926-022-10031-0)
Supplement: Supplementary file 1 — Supplementary file1 (DOCX 51 kb) [file 10926_2022_10031_MOESM1_ESM.docx]

**Appendix 1. Survey content for RCT authors**

**The RCT which we contacted you about and will be referring to from now on in this survey.**

**Title:**

**Abstract:**

**1. BASELINE CHARECTERISTICS**

Demographics:

- Country of residence *……..*

Professional background (tick all that apply)

- Physician
- Occupational physician
- Insurance physician
- Other physician, specialty: ………….
- Occupational therapist
- Physical therapist
- Psychologist
- Psychiatrist
- Epidemiologist
- Other: ………..
- Years experience in research with work outcomes: *(open field for numeric value)*
- Number of RCTs conducted or participated in over the last 5 years that measured work outcomes *(open field for numeric value)*

Role in RCT:

- First author
- Last author

What field is your expertise?

- Disease: …………….
- Intervention: …………..
- Economic evaluations: …..
- Other

**2. OUTCOMES**

**ⓘ**

***Definition of work participation***

*For the purpose of this survey we define work participation outcome as any outcome which is used to determine whether/to what extent a person is able/wants to participate in paid labour either with an employer or being self-employed. The outcomes may be either objectively measurable (e.g. sickness absence, presenteeism, productivity, paid work, occupational status, work ability) or based on subjective experience (e.g. motivation to work).*

**Outcome vs. measurement method**

Please note the difference between an outcome and measurement method. An outcome refers *ONLY to the construct*, e.g. work-ability, presenteeism, sick leave, return to work self-efficacy. *NOT to the way the constructs are measured.* Questions about measurement instruments (e.g. questionnaires, registers, routinely collected data) or psychometric qualities of measurement instruments (reliability, validity, responsiveness) will follow in a separate section.

1. Did you include work participation outcomes as a primary outcome in the RCT?

- Yes
- No

2. How did you choose the work participation outcome(s) for the RCT?

*(Mandatory Yes/No selection for every closed-ended answer. Mandatory to fill in the open field for a checked box that requires specifications)*

YES/NO

- We selected work outcome(s) used in similar studies by other authors
- We selected work outcome(s) which were previously used by me or my research group
- We selected work outcome(s) based on the anticipated impact of the intervention(s) (i.e. outcomes tailored to the intervention(s))
- We selected work outcome(s) based on the relevance to the study population
- We had pre-specified criteria for which type of measurement methods would be acceptable and this influenced my choice for a specific outcome (definition
- The work outcomes were based on a consensus process (e.g. Delphi study) of experts or an advisory group
- We consulted patients and/or consumers which outcomes are most relevant for the study population
- We consulted stakeholders other than patients or consumers on which outcomes are more relevant for the study population
- For selecting work outcomes(s)we took a specific perspective (e.g. health, economic, employer or societal perspective), namely …
- For selecting work outcome(s) I used a theoretical framework / conceptual model / logic model, namely …
- We used a core outcome set, specify ………
- Other …

3. How did you decide which outcome should be primary, secondary or tertiary?

*(Mandatory Yes/No selection for every closed-ended answer. Mandatory to fill in the open field for a checked box that requires specifications)*

YES/NO

- We did not define any primary (or secondary) outcomes
- We used a core outcome set in which this was defined, namely …
- We created a logic model of intervention components, treatment pathways, outputs and outcomes. The logic model showed which outcomes would be proximal, distal, or surrogate and which factors could be mediating or confounding. This determined which outcomes should be primary or secondary.
- The choice for the primary outcome was determined by taking a specific perspective (e.g. health, economic, employer or societal perspective), namely….
- The content validity of measurement instruments played a role in determining which outcomes should be primary/secondary
- The reliability of measurement instruments played a role in determining which outcomes should be primary/secondary
- The utility of measurement instruments played a role in determining which outcomes should be primary/secondary
- We described in a protocol which outcomes would be primary, secondary or tertiary
- We described in a protocol the analysis plan for primary and secondary outcomes

4. Pre-specifying outcomes

*(Yes/No selection mandatory)*

YES/NO

- We pre-specified all outcomes in a protocol, and analyzed and reported the results on these outcomes as planned
- Some or all outcomes were not pre-specified but explored post-hoc (for example in (sub)group analyses)

5. Would you choose to use the same work outcomes in the future?

- Yes
- No, why not? ….

6. Do you have suggestions for work outcomes that should be included in a similar RCTs?

- Yes, specify …
- No

7. Do you have suggestions for generic work outcomes that should always be included RCTs?

- Yes, specify …
- No

**MEASUREMENT METHODS**

*(Mandatory Yes/No selection for every closed-ended answer. Mandatory to fill in the open field for a checked box that requires specifications)*

YES/NO

- We described the measurement method in a protocol (e.g. questionnaires, registers, routinely collected data)
- It was important to use an instrument with high (content) validity;…. *(optional open field)*
- It was important to use an instrument with high reliability;…. *(optional open field)*
- It was important to use an instrument with high responsiveness;…. *(optional open field)*
- It was important to use an instrument with good utility/interpretability; … *(optional open field)*
- We wanted to use an instrument that could be used at baseline as well as at follow up
- We chose an instrument that could show composite outcomes; …. *(optional open field)*
- We added created a self-developed item(s) because; …. *(optional open field)*
- We added a self-developed item(s) to an existing questionnaire because; …. *(optional open field)*
- A legislative or (social) insurance scheme factors determined the choice for the measurement method, ….*(optional open field)*
- We found the following instrument(s)/method most favorable for measuring work outcomes …… because: …
- We find the following instrument(s) least favorable for measuring work outcomes …. because: …

Indicate below what you consider to be advantages and disadvantages of different types of measurement methods

|  | Patient reported outcome measures | Data from administrative registries (on sickness absence) | Data on disability pensions (for determining return to work) | Routinely collected data (e.g interview, clinicians report, phone call) |
| --- | --- | --- | --- | --- |
| Advantages |  |  |  |  |
| Disadvantages |  |  |  |  |

**TIMING AND FOLLOW-UP MEASUREMENTS**

*(Mandatory Yes/No selection for every closed-ended answer. Mandatory to fill in the open field for a checked box that requires specifications)*

YES/NO

- We pre-specified in a protocol why certain follow-up times were chosen
- When deciding the follow up times I looked at similar studies
- We used more than one method for follow-up (e.g questionnaire and telephone call)
- We included end of treatment, short term and long term time points
- We measured effects with post trial follow up (after intervention was done)
- It was not feasible/useful to do long term post trial follow up, because; ….
- We would find it important to measure post trial follow up, but could not do it due to restrictions in finance or otherwise; ..
- We think data from registries would be most preferred for long term follow up, because:
- We think patient reported data would be most preferred for follow up, because:

The ideal duration for follow-up time of *work status* relating outcomes should be:

*(Mandatory Yes/No selection for every closed-ended answer. Mandatory to fill in the open field for a checked box that requires specifications)*

YES/NO

- One month
- Three months
- Six months
- Twelve months
- Twenty four months
- More than 24 months
- No opinion
- It depends on something specific, namely:

The ideal duration for follow-up time of *sickness absence* relating outcomes should be:

YES/NO

- One month
- Three months
- Six months
- Twelve months
- Twenty four months
- More than 24 months
- No opinion
- It depends on something specific, namely:

The ideal duration for follow-up time of *work ability/ work functioning* relating outcomes should be:

YES/NO

- One month
- Three months
- Six months
- Twelve months
- Twenty four months
- More than 24 months
- No opinion
- It depends on something specific, namely:

The ideal duration for follow-up time of *presenteeism* relating outcomes should be:

YES/NO

- One month
- Three months
- Six months
- Twelve months
- Twenty four months
- More than 24 months
- No opinion
- It depends on something specific, namely:

The ideal duration for follow-up time of *employability (i.e. readiness for work, job seeking skills, work motivation)* related outcomes should be:

YES/NO

- One month
- Three months
- Six months
- Twelve months
- Twenty four months
- More than 24 months
- No opinion
- It depends on something specific, namely:

**3. Cos for Work**

**ⓘ** *A Core Outcome Set (COS) is a minimal set of outcomes which is to be used in all trials in a specific health field. Use of COS makes data pooling possible and decreases outcome reporting bias. The core outcomes are determined by stakeholders by means of a consensus process. Once it is agreed “what” should be measured in all trials as a minimum, the developers of the core outcome set look for the best possible way of “how” to measure such outcomes.*

*COS for Work will result in a universal set of work participation outcomes which are applicable for any type of intervention or health problem. The PICO for such trials:*

*P: people of the working age with any type of health problem who are either employed or want to work*

*I: any type of intervention (medical, vocational, behavioural, workplace)*

*C: any type of control*

*O: an outcome which indicate that a person is able to (re)gain work or is able to maintain working (optimally)*

*For more information please visit: www.cosforwork.org*

1. Which advantages do you see for a generic COS for work participation?

*(Mandatory to fill in the open field)*

……..

2. What do you expect could be barriers for the development and implementation of a generic COS for work and do you have any recommendations?

*(Mandatory Yes/No selection for every closed-ended answer. Mandatory to fill in the open field for a checked box that requires specifications)*

YES/NO

- International differences in social benefit and health care systems may require very different effect measures
- In my health field it is not possible to use a measurement instrument which is not disease specific
- In my health field work related definitions, outcomes, i.e. “what” should be measured is decided and cannot be revised
- There are disease specific core outcome sets which have included work outcomes and I would choose recommended outcomes from such a COS rather than from a generic COS for work
- Finding/creating clinimetrically sound measurement instruments with international cross-cultural validity may not be feasible
- A broad COS for work would have to involve too many stakeholders for reaching consensus
- It may be difficult to involve “patient’ representatives in the consensus process as the domain for this COS is already chosen and the focus of the consensus will evolve around methodological aspects of measuring work participation
- Other barriers: ……
- Any recomendations: …..

3. Should any of your previously used outcomes or measurement instruments NOT be considered for a COS for work?

- Yes, specify …
- No

4. In my view a COS for work should include at least:

*(Mandatory Yes/No selection for every closed-ended answer. Mandatory to fill in the open field for a checked box that requires specifications)*

Outcomes which measure:

- Work status
- Absenteeism
- Presenteeism
- Work ability/productivity
- Employability
- Other, specify ….

5. Being an author of trials which measure work participation you are a potential stakeholder/end-user of COS for Work. Would you be interested in participating in the consensus process on which work participation outcomes would be essential for COS for Work?

- Yes and I may be approached
- No

**Appendix 2. Survey content for systematic reviewers**

**1.BASELINE CHARECTERISTICS**

Demographics:

- Country of residence *……..*

Professional background (selecting more than one box possible)

- Physician
- Occupational physician
- Insurance physician
- Other physician, specialty: ………….
- Occupational therapist
- Physical therapist
- Psychologist
- Psychiatrist
- Epidemiologist
- Other: ………..
- Number of years experience in research with work outcomes: *(open field for numeric value)*
- Number of reviews conducted or participated in over the last 5 years that measured work outcomes *(open field for numeric value)*

Role in review:

- First author
- Co-author
- Principal/Chief Investigator

What field is your expertise?

- Disease: …..
- Intervention: ……
- Economic evaluations: …..
- Other …

**2. OUTCOMES**


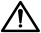


***Definition of work participation***

*For the purpose of this survey we define work participation outcome as any outcome which is used to determine whether/to what extent a person is able/wants to participate in paid labour either with an employer or as self-employed. The outcomes may be either objectively measureable (e.g. sickness absence, presenteeism, productivity, paid work, occupational status, work ability) or based on subjective experience (e.g. motivation to work).*

**Outcome vs. measurement method**

Please note the difference between an outcome and measurement method. An outcome refers *ONLY to the construct*, e.g. work-ability, presenteeism, sick leave, return to work self-efficacy. *NOT to the way the constructs are measured, e.g. measurement instruments or psychometric qualities.* Questions on the measurement methods will follow in a separate section.

1. How did you choose the work participation outcome(s) which were reported in the systematic review?

*(Mandatory Yes/No selection for every closed-ended answer. Mandatory to fill in the open field for a checked box that requires specifications)*

- We selected work outcome(s) used in similar studies by other authors
- We selected work outcome(s) which were previously used by me or my research group
- We selected work outcome(s) based on the anticipated impact of the intervention(s) (i.e. outcomes tailored to the intervention(s))
- We selected work outcome(s) based on the relevance to the study population
- We had pre-specified criteria for which type of measurement methods would be acceptable and this influenced my choice for a specific outcome (definition
- The work outcomes were based on a consensus process (e.g. Delphi study) of experts or an advisory group
- We consulted patients and/or consumers which outcomes are most relevant for the study population
- We consulted stakeholders other than patients or consumers on which outcomes are more relevant for the study population
- For selecting work outcomes(s)we took a specific perspective (e.g. health, economic, employer or societal perspective), namely …
- For selecting work outcome(s) I used a theoretical framework / conceptual model / logic model, namely …
- We used a core outcome set, specify ………
- Other …

2. Pre-specifying outcomes

*(Yes/No selection mandatory)*

YES/NO

- We pre-specified all outcomes in a protocol and reported results on these outcomes as planned
- These were outcomes which were not pre-specified but explored post-hoc (for example in (sub)group analyses)

3. Would you choose to use the same work outcomes in the future?

*(Mandatory Yes/No selection for every closed-ended answer. Mandatory to fill in the open field for a checked box that requires specifications)*

- Yes
- No, why not? ….

4. Which of the following steps did you undertake to make a distinction in primary, secondary, tertiary outcomes?

*(Mandatory Yes/No selection for every closed-ended answer. Mandatory to fill in the open field for a checked box that requires specifications)*

- We did not define any primary (or secondary) outcomes
- We used a core outcome set in which this was defined, namely …
- We created a logic model of intervention components, treatment pathways, outputs and outcomes. The logic model showed which outcomes would be proximal, distal, or surrogate and which factors could be mediating or confounding. This determined which outcomes should be primary or secondary.
- The choice for the primary outcome was determined by taking a specific perspective (e.g. health, economic, employer or societal perspective), namely….
- The content validity of measurement instruments played a role in determining which outcomes should be primary/secondary
- The reliability of measurement instruments played a role in determining which outcomes should be primary/secondary
- The utility of measurement instruments played a role in determining which outcomes should be primary/secondary
- We described in a protocol which outcomes would be primary, secondary
- or tertiary
- We described in a protocol the analysis plan for primary and secondary outcomes

5. Do you have any suggestions for important additional work outcomes that should be included in a similar review?

- Yes, specify …
- No

**DATA SYNTHESIS**

1. We pre-specified which type of measurement instruments/methods would be eligible for data synthesis in a protocol.

YES/NO


2. We experienced trouble with comparing/pooling data from RCTs.

YES/NO


3. If yes, what were the issues you came across?

*(Mandatory Yes/No selection for every closed-ended answer. Mandatory to fill in the open field for a checked box that requires specifications)*

- Different definitions of RTW (i.e. time until first day, full/part time employment, return to own/adjusted/new job, variety minimal important change in durable RTW, etc.), namely: ……..
- Different definitions of work status, namely: ……..
- Varying definitions of workability/work functioning/ productivity, namely: ……
- Combining self-reported data and registry based data
- Different ways of measuring sickness absence (days, hours, weeks missed)
- Different cut-off points and follow-up times
- Other ..

4. Indicate below what you consider to be advantages and disadvantages of different types of measurement methods

|  | Patient reported outcome measures | Data from administrative registries (on sickness absence) | Data on disability pensions (for determining return to work) | Routinely collected data (e.g interview, clinicians report, phone call) |
| --- | --- | --- | --- | --- |
| Advantages |  |  |  |  |
| Disadvantages |  |  |  |  |

5. Ideally RCTs should report

*(Mandatory Yes/No selection for every closed-ended answer. Mandatory to fill in the open field for a checked box that requires specifications)*

*For work status* relating outcomes:

- Mean differences
- Odds ratio’s
- Standardized mean differences
- Duration of employment in days
- Duration of emplpyment in weeks
- Other: ….

For *sickness absence/ return to work* relating outcomes:

- Sick leave spells
- Number of persons as sick listed
- Percentage of workers as sick listed
- Sick leave duration in work days
- Sick leave duration in work weeks
- Other: ….

For *work ability/ work functioning/ productivity* relating outcomes:

- Mean differences
- Odds ratio’s
- Standardized mean differences
- Other: ….

**FOLLOW-UP TIMES**

The ideal duration for follow-up time in RCTs of *work status* relating outcomes should be:

*(Mandatory Yes/No selection for every closed-ended answer. Mandatory to fill in the open field for a checked box that requires specifications)*

YES/NO

- One month
- Three months
- Six months
- Twelve months
- Twenty four months
- More than 24 months
- No opinion
- It depends on something specific, namely:

The ideal duration for follow-up time in RCTs of *sickness absence* relating outcomes should be:

- One month
- Three months
- Six months
- Twelve months
- Twenty four months
- More than 24 months
- No opinion
- It depends on something specific, namely:

The ideal duration for follow-up time in RCTs of *work ability/ work functioning*  relating outcomes should be:

- One month
- Three months
- Six months
- Twelve months
- Twenty four months
- More than 24 months
- No opinion
- It depends on something specific, namely:

The ideal duration for follow-up time in RCTs of *presenteeism* relating outcomes should be:

- One month
- Three months
- Six months
- Twelve months
- Twenty four months
- More than 24 months
- No opinion
- It depends on something specific, namely:

The ideal follow-up time in RCTs of *employability (i.e readiness for work, job seeking skills, work motivation)* related outcomes should be:

- One month
- Three months
- Six months
- Twelve months
- Twenty four months
- More than 24 months
- No opinion
- It depends on something specific, namely

**3. Cos for Work**

**ⓘ** *A Core Outcome Set (COS) is a minimal set of outcomes which is to be used in all trials in a specific health field. Use of COS makes data pooling possible and decreases outcome reporting bias. The core outcomes are determined by stakeholders by means of a consensus process. Once it is agreed “what” should be measured in all trials as a minimum, the developers of the core outcome set look for the best possible way of “how” to measure such outcomes.*

*COS for Work will result in a universal set of work participation outcomes which are applicable for any type of intervention or health problem. The PICO for such trials:*

*P: people of the working age with any type of health problem who are either employed or want to work*

*I: any type of intervention (medical, vocational, behavioural, workplace)*

*C: any type of control*

*O: an outcome which indicate that a person is able to (re)gain work or is able to maintain working (optimally)*

*For more information please visit: www.cosforwork.org*

1. Which advantages do you see for a generic COS for work participation?

*(Mandatory to fill in the open field)*

……..

2. What do you expect could be barriers for the development and implementation of a generic COS for work and do you have any recommendations?

*(Mandatory Yes/No selection for every closed-ended answer. Mandatory to fill in the open field for a checked box that requires specifications)*

YES/NO

- International differences in social benefit and health care systems may require very different effect measures
- In my health field, it is not possible to use a measurement instrument which is not disease specific
- In my health field ,work related definitions, outcomes, i.e. “what” should be measured is decided and cannot be revised
- There are disease specific core outcome sets which have included work outcomes and I would choose recommended outcomes from such a COS rather than from a generic COS for work
- Finding/creating measurement instruments with international cross-cultural validity may not be feasible
- A broad COS for work would have to involve too many stakeholders for reaching consensus
- It may be difficult to involve “patient’ representatives in the consensus process as the domain for this COS is already chosen and the focus of the consensus will evolve around methodological aspects of measuring work participation
- Other barriers: ……
- Any recomendations: …..

3. Should any of your previously used outcomes or measurement instruments NOT be considered for a COS for Work?

- Yes, specify …
- No

4. In my view a COS for work should include:

*(Mandatory Yes/No selection for every closed-ended answer. Mandatory to fill in the open field for a checked box that requires specifications)*

Outcomes which measure:

- Work status
- Absenteeism
- Presenteeism
- Work ability/productivity
- Employability
- Other, specify ….

5. Being an author of review which evaluate work participation you are a potential stakeholder/end-user of COS for Work. Would you be interested in participating in the consensus process on which work participation outcomes would be essential for COS for Work?

- Yes, and I may be approached
- No

**Appendix 3. Considerations to make a work participation outcomes the primary outcome.**

Questions were answered by 26 reviewers and 64 authors. Closed-ended answer options were “Yes” or “No”. Here, we report the respondents who agreed with the proposed statement.

| **Reasons for choosing primary/secondary outcomes were:** | **Agreement with the proposed statement** | |
| --- | --- | --- |
| **N=90 unless indicated othewise** | **RCT Authors**  **% (n)** | **Reviewers   % (n)** |
| The validity of instruments played a role | 48% (30) | **-** |
| There was no distinction between primary and secondary WPOs | 44% (28) | **-** |
| The reliability of measurement instruments played a role | 43% (27) | **-** |
| Primary outcome was the most feasible for sample size calculation | 33% (21) | **-** |
| The primary outcome was determined by stakeholders | 28% (18) | 19% (5) |
| The choice for a primary or secondary WPO was based on a COS (n=89) | 16% (10) | 4% (1) |
| A logic model determined choice | 11% (7) | 12% (3) |

- the question was not asked in this survey for this group of respondents; ; RCT – randomized controlled trial; WPO – work participation outcome; COS – core outcome set

**Appendix 4. Pooling work participation outcomes in systematic reviews**

| **Personal approaches and experience with pooling work participation outcomes**  N=25 | **Reviewers who agreed with statement**  **%, (n)** |
| --- | --- |
| The measurement instruments/methods that were eligible for comparison were pre-specified by the reviewers | 84% (21) |
| The reviewer experienced challenges when pooling data on work participation outcomes | 72% (18) |
| Experienced problems with: |  |
| - varying cut-off points, follow-up times | 80% (20) |
| - heterogeneous definitions of RTW (i.e. time until first day, full/part time employment, return to own/adjusted/new job) | 56% (14) |
| - sickness absence data/definitions | 44% (11) |
| - work status outcomes data/definitions | 36% (9) |
| - combining self-report and registry data | 24% (6) |
| - productivity outcomes data/definitions | 20% (5) |
| - other kind of problem with data synthesis | 4% (1) |
| **Preferred statistic for individual work participation outcomes in general** |  |
| Work status |  |
| - Duration of employment in work days | 68% (17) |
| - Confidence intervals | 56% (14) |
| - Mean difference between arms | 52% (13) |
| - Odds ratios | 48% (12) |
| - Standardized mean differences | 44% (11) |
| - Other suggestions (open field) | 24% (7) |
| - Duration of employment in weeks | 20% (5) |
| Absenteeism |  |
| - Sick leave duration in work days | 84% (21) |
| - Number of persons as sick listed | 52% (13) |
| - Sick leave spells | 40% (10) |
| - Percentage of persons as sick listed | 36% (9) |
| - Sick leave duration in work weeks | 20% (5) |
| - Sustainable return to work (e.g. no sickness absence for amount of time) | 16% (4) |
| - other suggestions (open field) | 16% (4) |
| At-work productivity loss |  |
| - Mean and standard deviation | 56% (14) |
| - Mean differences | 52% (13) |
| - Standardized mean differences | 48% (12) |
| - Confidence intervals | 44% (11) |
| - Odds ratios | 28% (7) |
| - Median and interquartile range | 24% (6) |
| - Other suggestions (open field) | 16% (4) |
